# A survey of primary care physician practices in antibiotic prescribing for the treatment of uncomplicated male gonoccocal urethritis

**DOI:** 10.1186/1471-2296-12-35

**Published:** 2011-05-18

**Authors:** Alessandra Falchi, Andrea Lasserre, Anne Gallay, Thierry Blanchon, Patrice Sednaoui, François Lassau, Veronique Massari, Clément Turbelin, Thomas Hanslik

**Affiliations:** 1 INSERM, UMPC UMR-S 707, F-75012, Paris, France; 2 UPMC Université Paris 06, UMR-S U707, F-75012, Paris, France; 3 Institut de Veille Sanitaire, St Maurice, Paris, France; 4 Institut Alfred Fournier Paris, France; 5 Hôpital Saint-Louis, AP-HP, Paris, France; 6 Université Versailles Saint Quentin en Yvelines, F-78000, Versailles, France; 7 Assistance Publique Hôpitaux de Paris, Service de Médecine Interne, Hôpital Ambroise Paré, F-92100, Boulogne Billancourt, France

## Abstract

**Background:**

The development of resistance to antimicrobial therapy by *Neisseria gonorrhoeae *causes on-going problems for individual case management of gonorrhoea. Surveillance data about *N. gonorrhoeae *have indicated an increase in the incidence of gonorrhoea in France in 2006. As a consequence of the development of antibiotic resistance in *N. gonorrhoeae*, French guidelines excluded fluoroquinolones as a standard treatment for *N. gonorrhoeae*. Ceftriaxone became the recommended treatment, associated with azithromycin for *Clamydia trachomatis *infection. Our aim was to describe the practice patterns of general practitioners (GPs) in managing the antibiotic treatment of patients with symptoms suggestive of uncomplicated male urethritis.

**Methods:**

We developed a clinical vignette describing a man with typical gonococcal urethritis symptoms to elicit questions about antibiotic treatment. We mailed the electronic questionnaire to a random sample of 1000 French GPs belonging to the *Sentinelles *Network.

**Results:**

By the end of the survey period, 350 vignettes were received, yielding a response rate of 35%. Sixty-six GPs (20.2%) prescribed the recommended antibiotics for the simultaneous treatment of *N*. *gonorrhoeae *and *C. trachomatis *infections, while 132 GPs (40.4%) prescribed only non-recommended antibiotics, including ciprofloxacin in 69 cases (21.1%). General practitioners with less than 10 years in practice showed better compliance to guidelines than those with more years in practice (p < 0.05).

**Conclusions:**

The results suggest a mismatch between the guidelines and the antibiotic treatment of male uncomplicated urethritis by French GPs, mostly among the subgroup of physicians who have been in practice longer. Educational approaches based on practice feedback need to be developed to improve these deficits in the quality of care.

## Background

Over the last few years, the number of gonorrhoea cases, caused by the gram negative bacterium *Neisseria gonorrhoeae*, has continually increased in many European countries, the United States and China [1-3]. However, over the past 60 years *N. gonorrhoeae *has developed resistance to multiple classes of antimicrobials. Since the first report of tetracycline resistance in 1985, gonococci that are resistant to tetracycline have spread globally [4]. During a sentinel surveillance study in Western Europe in 2004, a total of 1055 gonoccocal isolates were analysed of which 21.3% showed resistance to penicillin [5]. In addition, high levels of ciprofloxacin-resistant strains were reported worldwide in the early 1990s [6].

In France, an increase in the incidence of gonorrhoea cases has been indicated since 1998 [7]. In 2006, the number of gonorrhoea cases increased by more than 50% compared to 2005. Resistance of *N. gonorrhoeae *to ciprofloxacin sharply increased from 0.70% in 1998 to 43% in 2006 [7,8].

An essential element in gonorrhoea control is the availability and provision of appropriate, effective antimicrobial therapy. Effective treatment not only eradicates infection in the affected individual and prevents the development of complications, but it also has an important public health benefit of decreasing transmission and eliminating reservoirs of infection. Thus, improving the appropriateness of antimicrobial use for the treatment of gonorrhoea is an important goal at national and international levels. New guidelines rationalizing antibiotic prescription for the probabilistic treatment of uncomplicated male urethritis were published in France in 2005 by the French Health Products Safety Agency (Afssaps) [9], recommending ceftriaxone as the first-line treatment, always associated with azithromycin for *Clamydia trachomatis *infection. Briefly, the recommended regimens, for all adult and adolescent patients, are ceftriaxone (intravenous/intramuscular) or cefixime (oral dosage) only if injectable is refused or not possible to do. If the patient is allergic to ceftriaxone or cefixime an alternative regimen is spectinomycin. Recommended regimens for *C. trachomatis *are azytromicin or doxycycline. As a consequence of the development of antibiotic resistance in *N. gonorroheae*, new French guidelines excluded fluoroquinolones as a standard treatment for *N. gonorrhoeae*.

General Practitioners (GPs) play an important role in preventing and treating male gonoccocal urethritis [10]. Consequently, it is worth making sure that GPs have a good working knowledge of antimicrobial use for gonorrhoea treatment. In this study, we conducted a national cross-sectional study using a clinical vignette to examine GPs' antibiotic prescribing in the treatment of gonoccocal male urethritis.

## Methods

From June 2008 to August 2008, a cross-sectional study was conducted among GPs belonging to the Sentinelles Network [11], a computerized disease-surveillance system with about 1260 active volunteer GPs located throughout mainland France. The GPs participating in the Sentinelles Network are representative of the whole French GP population regarding age, location, type (rural/urban) and kind of practice (alone/pluridisciplinary) [12].

General practitioners who had previously agreed to participate at least once in our ad-hoc epidemiological studies by electronic means (n = 1000) were invited to complete a questionnaire

In September 2008, after data collection, a sample of 70 randomly selected non-respondent GPs was contacted by e-mail to ascertain the reasons for non-participation.

The French Sentinelles Network were given ethical approval (CNIL 471393) for the study. Moreover, the participation of GPs was voluntary and they could withdraw at any time. All data were handled confidentially and the results from the study were presented in a non-identifiable way.

### Survey development

A scientific committee composed of one specialist in venereal diseases, one epidemiologist, one general internist and one microbiologist developed a vignette [13,14] designed to examine current practice patterns in the antibiotic treatment of uncomplicated male urethritis using scientific literature and guidelines [9,13,14]. The vignette was developed using a standardized protocol [13,14]. This approach has been shown to be an effective and cost-efficient method for measuring physicians' clinical decision making [13,14]. A pilot study was conducted with eight volunteer GPs of the Sentinelles Network that validated the vignette.

The scientific committee defined a master criteria list to review GP responses using scientific literature and guidelines [9,13-15]. We submitted the candidate criteria to a panel of 12 academic and community physicians of various specialities (general practice, infectious diseases, venereal diseases, microbiologists and public health). Based on their recommendations and group consensus, we finalized a master criteria list. Responses were considered appropriate if the hypothetical patient was treated simultaneously for *N. gonorrhoeae *and *C. trachomatis *infections with the following recommended first-line antibiotics:

a) ceftriaxone (250 to 500 mg intravenously or intramuscularly, in a single dose) or cefixime (400 mg orally in a single dose) or, as an alternative regimen, spectinomycin (2 g intramuscularly in a single dose) for *N. gonorrhoeae *treatment

b) azithromycin (1 g orally in a single dose) or doxycycline (100 mg orally twice a day for 7 days) for *C. trachomatis *treatment.

The vignette was submitted to the GPs via a web-based electronic format (see additional file): the GPs were asked to write a description of their treatment plan in an open-ended format. A time constraint of 20 minutes was imposed for completing the vignette. After answering a given section, participants could not return to the previous section to revise their answers.

After the clinical vignette, a new section measuring the GPs' knowledge of French guidelines and of the increasing resistance to ciprofloxacin for gonorrhoea treatment through two questions completed the survey:

1. "Are you aware of the increasing rate of ciprofloxacin resistance for gonorrhoea?"

2. "Are you aware of the existence of the new guidelines for the management of urethritis?"

At the end of the study, each participating GP received an individual report of his results, the average performance of the group and the responses to the vignette.

### Statistical analysis

Data were analysed using the R software program (http://www.r-project.org) using descriptive statistics for frequencies and chi-square tests for the comparisons of independent proportions, with Fisher's tests where appropriate. The level of significance was set as p < 0.05.

The GPs' answers were reviewed by two independent reviewers. An inter-rater reliability analysis using the Kappa statistic was performed to determine consistency among the raters [16]. The inter-rater reliability for the raters was found to be Kappa = 0.83 (p < 0.001), suggesting a very good agreement.

## Results

### Sample

By the end of the survey period, 350 vignettes were received, yielding a response rate of 35%. The vignette was fully completed by 327 GPs (93.4%).

The responders were predominantly male (83.4%) with a mean age of 52 ± 7.5 years (min = 29; max = 69). Their practices were mainly located in urban zones (60%) (Table 1).

**Table 1:** Demographic characteristics of GP respondents, non-respondents and GPs of the Sentinelles Network

Demographic characteristics	GP respondents No = 350	GP non respondents No = 650	GPs of the Sentinelles Network No = 1260	p value
Sex (male) %	83.4%	82%	82%	0.97

Age (years), mean ± SD	52 ± 7.5	52.3 ± 7.6	52.8 ± 7.8	0.96

Practice location,%				

Urban	60%	61%	62%	0.96

Sub-Urban	19%	16%	18%	0.96

Rural	21%	19%	20%	0.94

Demographic characteristics (age, sex and location) of the GP responders, non-responders and of the GPs of the Sentinelles Network are shown in Table 1.

Among the 70 non-responding GPs, 67% (n = 47) declared that they had no experience of urethritis management in their practice, 26% (n = 18) did not participate because of a lack of time and 7% (n = 5) did not participate for other several reasons (e.g. holidays, the survey was too complex or they were not interested.

### Antimicrobial agents used

Sixty-six GPs (20.2%) prescribed the simultaneous administration of the recommended antibiotics for *N. gonorrhoeae *and *C. trachomatis *infections (Table 2) for the hypothetical patient.

**Table 2:** Antibiotic treatments prescribed by the GPs

**Treatments prescribed**	**Respondents No. (%)**
No = 327 (%)	
Two recommended antibiotics*	66 (20.2)
**One recommended antibiotic**	114 (34.8)
against *N. gonorrhoeae *^**Ψ**^	69 (21.1)
against *C. trachomatis *^***ΨΨ***^	45 (13.7)
**One non-recommended antibiotic**	147 (45)
Ciprofloxacin	69 (21.1)
Amoxicillin	24 (7.3)
Others	54 (16.6)

*for *N. gonorrhoeae and C. trachomatis *co-infections

^**Ψ **^*Ceftriaxone or spectinomycin or cefixime*

^***ΨΨ ***^*Azithromycin or doxycycline*

One hundred and fourteen (34.8%) GPs prescribed only one recommended antibiotic against *N. gonorrhoeae *(n = 69) or against *C. trachomatis *(n = 45). One hundred and thirty-two GPs (40.4%) prescribed only non-recommended antibiotics, including ciprofloxacin, in 69 cases (Table 2).

### Knowledge of fluoroquinolone resistance emergence and the treatment guidelines

When asked about the fluoroquinolone resistance of *N. gonorrheoae*, 26% (n = 84) of the respondents declared that they were aware of the high rate of resistance in France. The existence of the French guidelines was declared known by 25% (n = 81) of GPs. Among the 69 GPs who prescribed ciprofloxacin as first-line treatment, 84% (n = 58) were not aware of the emergence of fluoroquinolone resistance.

### Influence of prescriber factors

General practitioners who had been in practice for 10 years or less were more likely to prescribe the recommended antibiotics than GPs with more years in practice (p < 0.0001; Table 3). General practitioners who had been in practice for 10 years or less were also more likely to be aware of the high rate of fluoroquinolone resistance and of the existence of guidelines (p < 0.0001; Table 3). Gender and geographic location (urban, suburban or rural) of the GP was not associated with the treatments prescribed or knowledge of the guidelines (p > 0.05).

**Table 3:** Association between years in practice and the physicians' practice patterns in managing gonoccocal urethritis treatment and their knowledge of fluoroquinolone resistance emergence and of the guidelines

Years of practice	≤**10 years** No. (%)	11-20 years No. (%)	>20years No. (%)	p value
**First-line treatment**				

**One recommended antibiotic against *N. gonorrhoeae***^***¥***^	18 (36)	22 (28)	29 (15)	0.0012

**One recommended antibiotic against *C. trachomatis ***^***ΨΨ***^	12 (42)	19 (24)	14 (2.5)	0.0002

Two antibiotics prescribed concomitantly*	20 (40)	26 (32)	20 (10)	<0.0001

**Knowledge of fluoroquinolone resistance emergence and guidelines**				

**Awareness of the existence of the French guidelines**	22 (44)	26 (32.5)	33 (16.8)	<0.0001

**Awareness of fluoroquinolone resistance**	28 (56)	24 (30)	32 (16)	<0.0001

*for *N. gonorrhoeae and C. trachomatis *co-infection

^**Ψ **^*Ceftriaxone or spectinomycin or cefixime*

^***ΨΨ ***^*Azithromycin or doxycycline*

## Discussion

### Summary of the main findings

In this study, only a minority of the GPs (20.2%) correctly co-prescribed the first-line treatment recommended for *N. gonorrhoeae *and *C. trachomatis *co-infection. Moreover, the GPs frequently prescribed non-recommended first-line treatment, including ciprofloxacin, despite the high level of fluoroquinolone resistance of *N. gonorrhoeae *in France (43% during 2006) [7,8].

The relatively low level of prescribed treatment against *C. trachomatis *could be explained by the description of the purulent discharge of the urethritis in the vignette, which is a typical sign of *N. gonorrhoeae *infection. These results are in agreement with a previous study on the compliance of emergency department practitioners with the Centers for Disease Control (CDC) recommendations for diagnosing and treating sexually transmitted diseases. This study showed that the recommended antibiotic regimens for *N. gonorrhoeae *and *C. trachomatis *were correctly co-prescribed in only one third of cases [17].

In this study, GPs who had been in practice for 10 years or less were more likely to prescribe first-line treatment in accordance with the guidelines and they were better informed about the increasing rate of fluoroquinolone resistance and about the new national strategy for the therapeutic management of probabilistic treatment for uncomplicated male urethritis than physicians who had been in practice longer.

The results suggested that the overall rate of compliance with national guidelines concerning antibiotic choice must be improved among GPs. In particular, the subgroups of physicians who have been in practice for longer are at risk of providing lower-quality care.

These findings are consistent with other studies that suggested an inverse relationship between years in practice and the quality of care provided [18,19]. A systematic review which evaluated the relationship between clinical experience and performance suggested that physicians who have been in practice for a longer period and those who are older possess less factual knowledge, are less likely to adhere to appropriate standards of care, and may also have poorer patient outcomes. These effects seemed to persist in studies that adjusted for other known predictors of quality, such as patient comorbidity and physician volume or specialization [18].

As observed in this study, barriers to guideline adherence, such as a lack of agreement and a lack of familiarity, seem improbable in the case of urethritis, for which the recent guidelines are easily justified (a high rate of quinolone resistance) and quite straightforward to apply (a simple antibiotic prescription) [7-9]. One reason for the clinicians prescribing contrary to the guidelines is most probably unawareness of the guidelines. The fact that an increasing number of years in practice is associated with prescribing non-recommended antibiotics favours this hypothesis, although we cannot exclude other reasons. For example, GPs may find that prescribing an intramuscular treatment such as ceftriaxone might create a barrier because the patient has to buy the injectable drug and then go back to the GP to be injected with it. Another possible reason for little knowledge of the guidelines could be due to the volume of biomedical information available and the time needed to learn about new developments. The guidelines rationalizing antibiotic prescription for the probabilistic treatment of uncomplicated male urethritis were published in France in 2005 and disseminated to GPs at the national level by Afssaps [9], preferably by electronic means (mailing lists and publication on health websites). The fact that the recommendations were preferentially disseminated using electronic sources, of which younger GPs are greater users than older practitioners, could have added another barrier against compliance with the recommendations. It must be noted that, according to a survey by the Centers for Disease Control and Prevention's National Center for Health Statistics [20], it seems that a majority of office-based physicians use electronic medical record (EMRs) systems. A 2010 report by the Centers for Disease Control and Prevention's National Center for Health Statistics [20] reported that the younger the doctor, the more likely he or she was to embrace electronic medical records (EMRs), although the resistance to them by older physicians seems to have decreased as EMRs have become more common in offices and hospitals.

### Limitations of the study

There were several limitations to this study. The first was that we used a self-reported survey to measure compliance with guideline recommendations as it is possible that physicians answered questions in ways that reflected professional desirability rather than their own practices. The second limitation of this study was that we only proposed one scenario that did not cover the full clinical presentation of urethritis. However, the principal objective was to evaluate the application of guideline recommendations for the management and treatment of male uncomplicated urethritis, mostly due to gonorrhoea. Thirdly, there may have been limitations in generalizability to the French GP population, as reflected by the response rate of 35%. Moreover, the GPs who participated in this study were more concerned about urethritis than non-responders, as shown by the non-responders' answers, and therefore their practices might differ. However, we found no differences in the demographic data between responders and non-responders regarding age, gender, and location of the practice.

## Conclusions

### Implications for clinical practice and future research

This study suggests that interventions to improve physicians' knowledge about the management of public health issues are warranted, especially regarding the subgroup of physicians who have been in practice the longest. Moreover, because drug-resistant *N. gonorrhoeae *is becoming an increasing problem worldwide, such an evaluation of treatment practices would probably be useful in other countries.

## Competing interests

The authors declare that they have no competing interests.

## Authors' contributions

All authors read and approved the final manuscript. AF collected and analysed the data, conceived this paper and took primary responsibility for writing it. AL contributed to the study design, collected and analysed the data and reviewed the paper. AG was the principal investigator on the project and took part in the writing and review of the paper. TB contributed to the study design and reviewed the paper. PS contributed to the study design and reviewed the paper. FL contributed to the study design and reviewed the paper. VM contributed to the study design and reviewed the paper. CT contributed to the study design, designed the informatics support and reviewed the paper. TH conceptualized and wrote the proposal for the vignette, contributed to the study design and took part in the writing and review of the paper.

## Pre-publication history

The pre-publication history for this paper can be accessed here:

http://www.biomedcentral.com/1471-2296/12/35/prepub
